# U-Pb dating and geochemical dataset of fracture-filling calcite veins from the Bóixols-Sant Corneli anticline (Southern Pyrenees)

**DOI:** 10.1016/j.dib.2022.108636

**Published:** 2022-09-24

**Authors:** Daniel Muñoz-López, David Cruset, Jaume Vergés, Irene Cantarero, Antonio Benedicto, Vinyet Baqués, Xavier Mangenot, Richard Albert, Axel Gerdes, Aratz Beranoaguirre, Anna Travé

**Affiliations:** a Departament de Mineralogia, Petrologia i Geologia Aplicada, Facultat de Ciències de la Terra, Universitat de Barcelona (UB)^1^, Martí i Franquès s/n, Barcelona 08028, Spain; b Group of Dynamics of the Lithosphere (GDL), Geosciences Barcelona, GEO3BCN-CSIC, Lluís Solé i Sabarís s/n, Barcelona 08028, Spain; c UMR 8148 CNRS GEOPS, Université Paris-Saclay, Orsay 91405, France; d Caltech, Geological and Planetary Sciences, Pasadena, CA, USA; e Department of Geosciences, Goethe-University Frankfurt, Frankfurt am Main 60438, Germany; f Frankfurt Isotope and Element Research Center (FIERCE), Goethe-University Frankfurt, Frankfurt am Main, Germany; g Departamento de Mineralogía y Petrología, Universidad del País Vasco, Bilbao, Spain

**Keywords:** Fluid flow, Calcite veins, Fractures, Geochemistry, U-Pb geochronology, Bóixols-Sant Corneli anticline, Southern Pyrenees

## Abstract

U-Pb dating and geochemical analyzes (δ^18^O, δ^13^C, Δ^47^, ^87^Sr/^86^Sr and elemental composition) have been applied to fracture-filling calcite veins and host carbonates from the Bóixols-Sant Corneli anticline, which developed along the front of the Bóixols thrust sheet in the Southern Pyrenees. This robust dataset is used to determine: (i) the absolute timing of fracturing and mineralization from fluid flow; (ii) the age and duration of fold evolution; and (iii) the variations and implications of fluid behavior across the anticline, as has been described in the article “Spatio-temporal variation of fluid flow behavior along a fold: The Bóixols-Sant Corneli anticline (Southern Pyrenees) from U–Pb dating and structural, petrographic, and geochemical constraints – Marine and Petroleum Geology (2022) (Muñoz-López et al., 2022).

In this new contribution, we present the raw data that have been analyzed and discussed in the related research article and, also, the whole elemental and REE composition of calcite veins and host carbonates that has not been published yet. These data may be used to unravel the age and origin of veins, to understand their sequential evolution in orogenic belts and to compare our results with those obtained in similar settings worldwide.

**Specifications Table**

**Table:** 

Subject	Earth Sciences
Specific subject area	Geochemistry and Petrology
Type of data	Tables
How the data were acquired	U-Pb dating of calcite veins using laser ablation-inductively coupled plasma mass spectrometer (LA-ICP-MS). Carbon and oxygen isotopes of calcite veins and host carbonates with a Thermal Ionization Mass Spectrometer Thermo Electron MAT-252 (Thermo Fisher Scientific). Δ_47_ measurements of calcite veins with an automated acid digestion and gas purification device coupled to a dual inlet Thermo MAT253 Mass Spectrometer. ^87^Sr/^86^Sr ratios of calcite veins and host carbonates have been analyzed in a TIMS-Phoenix mass spectrometer (Isotopx). Elemental composition of calcite veins and host carbonates employing a magnetic sector field Element XR (HR-ICP-MS, high resolution inductively coupled plasma-mass spectrometer, Thermo Fisher Scientific).
Data format	Raw and analyzed
Description of data collection	The description of the data collection is presented in the Experimental design, materials, and methods section.
Data source location	Samples of calcite veins and related host rocks were collected at the Bóixols-Sant Corneli anticline (Southern Pyrenees). See coordinates of each sample in Table 1. Samples are stored at Facultat de Ciències de la Terra, Universitat de Barcelona (UB), Martí i Franquès s/n, Barcelona, 08028, Spain.
Data accessibility	Data within the article Repository Repository name: “Geochronological and geochemical data of calcite veins in the Bóixols-Sant Corneli anticline (Southern Pyrenees)” in Mendeley. Data identification number: 10.17632/sxhm5hghtw.1 Direct URL to data: https://data.mendeley.com/datasets/sxhm5hghtw/1
Related research article	Muñoz-López, D., Cruset, D., Vergés, J., Cantarero, I., Benedicto, A., Mangenot, X., Albert, R., Gerdes, A., Beranoaguirre, A., & Travé, A. (2022). Spatio-temporal variation of fluid flow behavior along a fold: The Bóixols-Sant Corneli anticline (Southern Pyrenees) from U–Pb dating and structural, petrographic and geochemical constraints. Marine and Petroleum Geology, 143, 105788. 10.1016/j.marpetgeo.2022.105788

## Value of the Data

• We present geochronological and geochemical data from fracture-filling calcite veins and carbonate host rocks from the outstanding exposed Bóixols-Sant Corneli anticline, along the front of the Bóixols thrust sheet (Southern Pyrenees). This robust dataset has been used to constrain the sequence of deformation, the age and duration of fold evolution, and the fluid flow behavior across the anticline.
• We include the raw data that have been analyzed and discussed in the related research article. We also include the whole elemental and REE composition of calcite veins and host carbonates that has not been published yet.
• These data are useful for geoscientists working on carbonate geochemistry and geochronology applying novel techniques such as U-Pb dating and clumped isotope thermometry.
• These data can be further used to determine the timing and thermal conditions of vein development during deformation and to compare our results with those obtained in similar settings worldwide.

## 1 Data Description

**Fig. 1:**
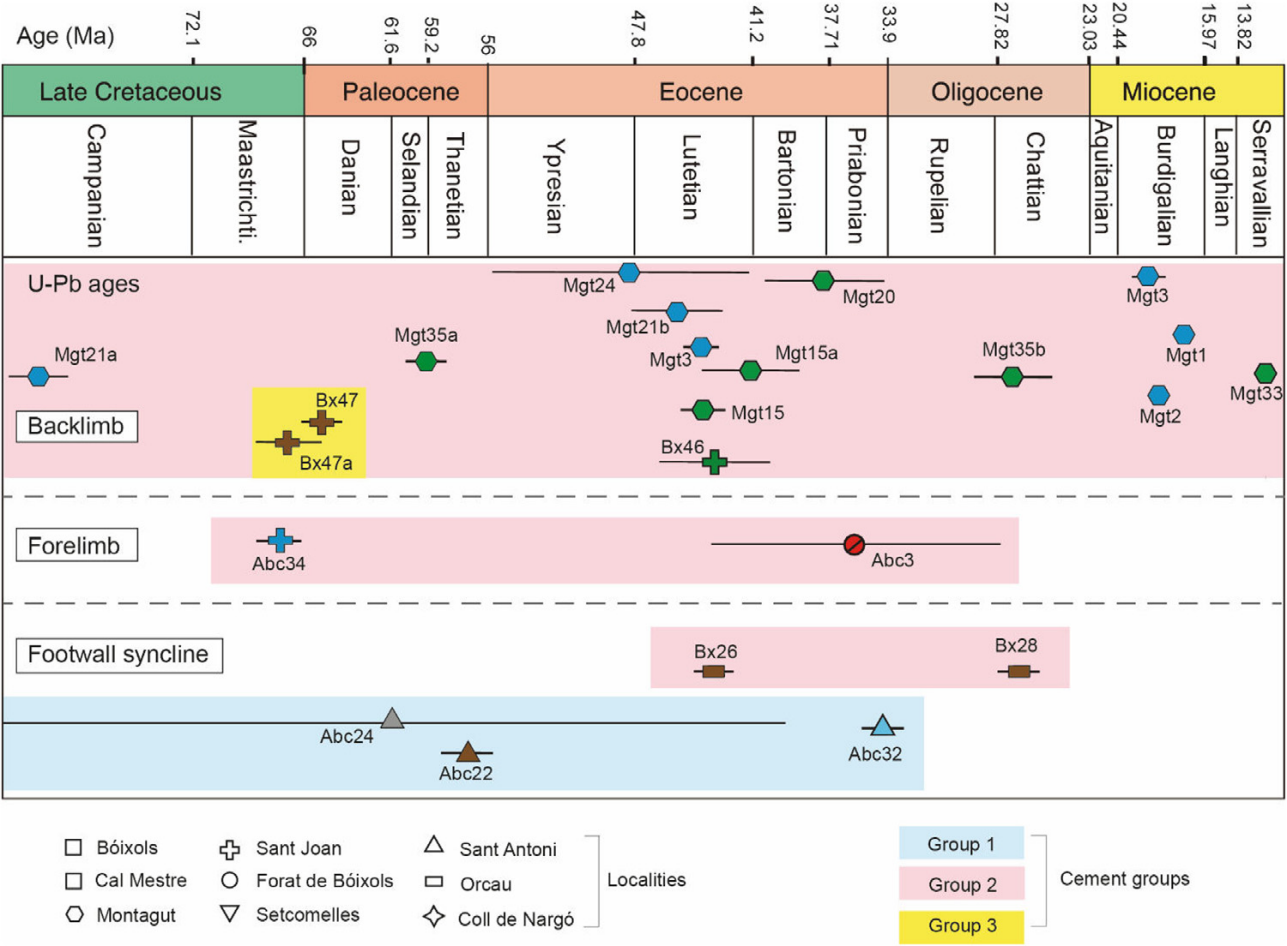
U-Pb ages of the different fracture-filling calcite veins in the Bóixols-Sant Corneli anticline. Ages are arranged in chronological order and according to the structural position of the anticline (i.e., backlimb, forelimb, footwall syncline). For homogeneity reasons, colors and symbols are those of [1].

**Fig. 2:**
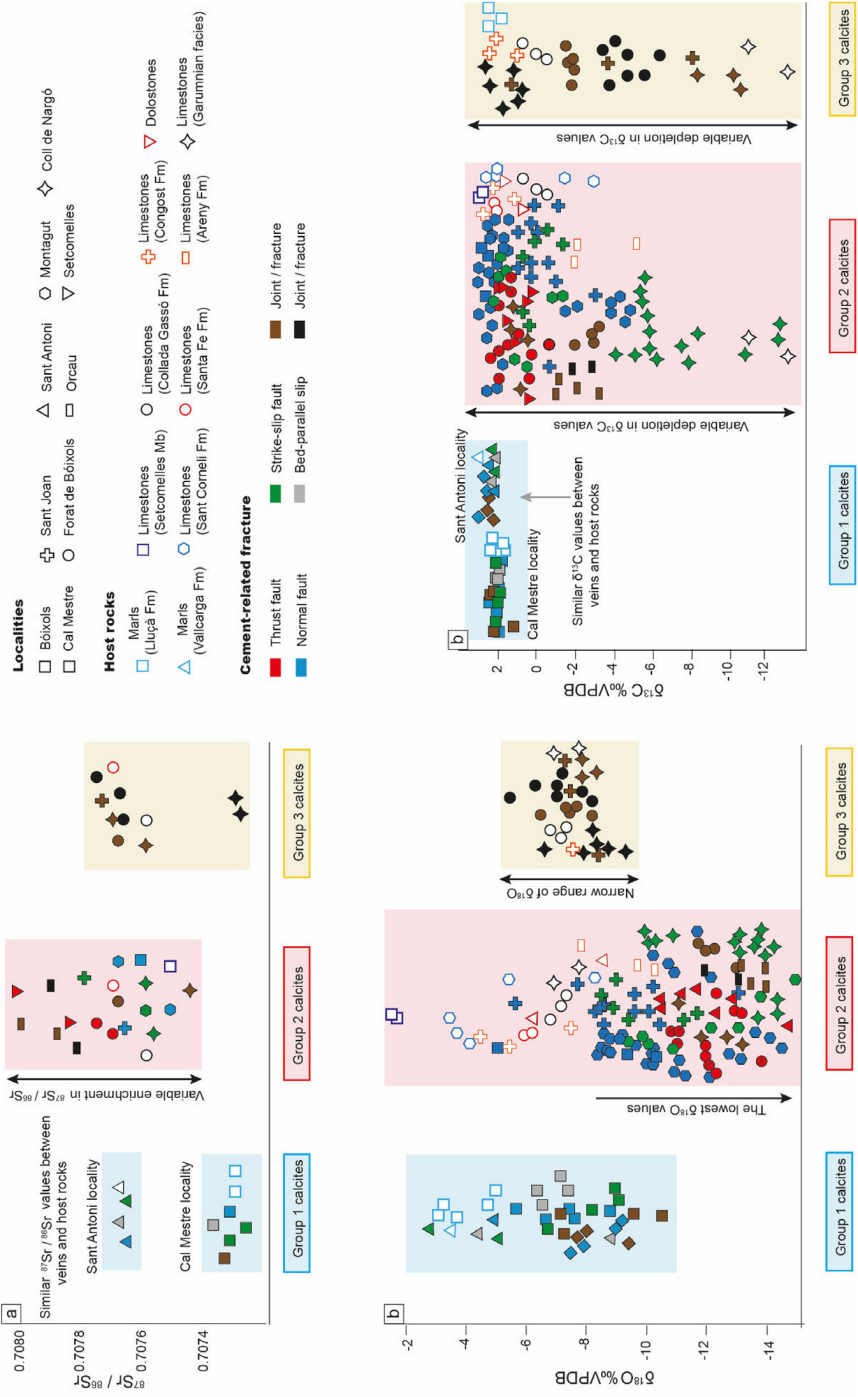
Geochemical results including (a) ^87^Sr/^86^Sr, (b) δ^18^O, and (c) δ^13^C of the different fracture-filling calcite cements and host rocks. The several symbols refer to localities where samples were taken, solid symbols refer to calcite cements and open symbols represent their related host rocks.

Geochronological and geochemical data of fracture-filling calcite cements and carbonate host rocks exposed in the Bóixols-Sant Corneli anticline (Southern Pyrenees) are presented here. Samples were collected in ten representative localities that cover the different fracture networks as well as the sedimentary successions involved in the formation of the anticline. The location and description of samples are shown in Table 1, the geochronological results in Table 2 and Fig. 1, and the geochemical dataset in Tables 3, 4 and Fig. 2. The complete elemental and isotopic composition of veins cements and host rocks is found in the Repository. The main features of fractures, and the detailed petrographic characteristics of vein cements and host rocks is found in [1] and elsewhere in [2,3].

**Table 1:** Location and description of the studied samples (calcite veins and host rocks) from the Bóixols-Sant Corneli anticline.

Locality	Description	Sample	Latitude	Longitude
Forat de	Damage zone (footwall)	Bx1 - Bx4, Bx35a - Bx36	42° 09′ 57.75′' N	1° 09′ 36.09′' E
Bóixols	Thrust fault	Bx5 - Bx6, Bx65 - Bx66	42° 09′ 57.12′' N	1° 09′ 39.10′' E
(FB)	Damage zone (hanging wall)	Bx7 - Bx8	42° 09′ 57.41′' N	1° 09′ 38.82′' E

Bóixols	Normal fault	Bx9 - Bx10, Bx37	42° 10′ 08.40′' N	1° 09′ 46.93′' E
(B)	Host rock	Bx38	42° 10′ 08.47′' N	1° 09′ 46.92′' E

	Strike slip faults	Bx11, Bx13	42° 10′ 25.65′' N	1° 09′ 54.80′' E
	Normal faults	Bx12, Bx14, Bx15	42° 10′ 25.47′' N	1° 09′ 54.18′' E
Cal Mestre	Bed-perpendicular veins	Bx16, Bx17	42° 10′ 24.90′' N	1° 09′ 55.45′' E
(CM)	Bed-parallel veins	Bx18, Bx39	42° 10′ 24.96′' N	1° 09′ 55.22′' E
	Strike slip faults	Bx40	42° 10′ 24.58′' N	1° 09′ 54.15′' E

	Strike slip faults	Bx19 - Bx21, Bx46	42° 11′ 45.68′' N	1° 10′ 35.81′' E
	Host rock	Bx45	42° 11′ 45.56′' N	1° 10′ 35.90′' E
	Vein	Bx47	42° 11′ 45.60′' N	1° 10′ 35.93′' E
	Strike slip fault	Bx48	42° 11′ 45.26′' N	1° 10′ 36.43′' E
Sant Joan	Normal faults	Bx49 - Bx53	42° 11′ 48.78′' N	1° 10′ 36.92′' E
(SJ)	Normal faults	Bx60 - Bx62	42° 11′ 44.71′' N	1° 10′ 36.07′' E
	Host rock	Bx63	42° 11′ 42.30′' N	1° 10′ 36.97′' E
	Normal fault	Bx64	42° 11′ 40.29′' N	1° 10′ 55.20′' E
	Normal fault	Bx65	42° 11′ 45.11′' N	1° 11′ 26.04′' E

	Veins	Bx22 - Bx27	42° 09′ 59.39′' N	0° 59′ 12.38′' E
Orcau	Vein	Bx28	42° 09′ 49.30′' N	0° 59′ 08.08′' E
(OC)	Vein	Bx29	42° 10′ 27.81′' N	0° 58′ 07.47′' E

	Normal faults	Bx32, Bx34	42° 11′ 27.75′' N	0° 57′ 29.22′' E
Sant Antoni	Strike slip fault	Bx33	42° 11′ 29.84′' N	0° 57′ 27.52′' E
(SA)	Bed-parallel vein	Bx35B	42° 11′ 29.90′' N	0° 57′ 27.55′' E
	Host rock	Bx42	42° 11′ 29.20′' N	0° 57′ 27.32′' E

Setcomelles (SET)	Thrust fault	Bx68 - Bx69	42° 10′ 57.10′' N	1° 12′ 23.20′' E

	Thrust fault	Abc1 - Abc4	42° 09′ 44.20′' N	1° 05′ 38.46′' E
	Veins	Abc5 - Abc6	42° 09′ 43.52′' N	1° 05′ 38.53′' E
	Thrust fault	Abc7 - Abc9	42° 09′ 44.57′' N	1° 05′ 38.20′' E
	Strike slip fault	Abc10	42° 09′ 44.06′' N	1° 05′ 38.33′' E
	Strike slip faults	Abc13 - Abc17	42° 09′ 41.71′' N	1° 05′ 34.21′' E
	Veins	Abc18 - Abc19	42° 09′ 40.72′' N	1° 05′ 32.73′' E
Abella de la	Strike slip fault	Abc20	42° 09′ 40.12′' N	1° 05′ 25.04′' E
Conca	Bed-perpendicular veins	Abc21 - Abc23	42° 09′ 36,37′' N	1° 05′ 55.74′' E
(ABC)	Bed-parallel vein	Abc24	42° 09′ 36,37′' N	1° 05′ 55.74′' E
	Veins	Abc25 - Abc27	42° 09′ 27.44′' N	1° 05′ 53.06′' E
	Strike slip faults	Abc28 - Abc30	42° 09′ 24.50′' N	1° 05′ 54.25′' E
	Normal faults	Abc31 - Abc33	42° 09′ 36.37′' N	1° 05′ 55.74′' E
	Normal faults	Abc34 - Abc35	42° 09′ 42.41′' N	1° 05′ 49.64′' E

	Normal faults	Mgt 1 – Mgt 3	42° 11′ 50.59′' N	1° 02′ 13.75′' E
	Normal faults	Mgt 4 – Mgt 6	42° 11′ 50.78′' N	1° 02′ 14.14′' E
	Host rock	Mgt 7	42° 11′ 51.14′' N	1° 02′ 14.09′' E
	Normal faults	Mgt 8 – Mgt 9	42° 11′ 53.80′' N	1° 02′ 11.15′' E
	Normal faults	Mgt 10 – Mgt 12	42° 11′ 53.76′' N	1° 02′ 09.98′' E
Montagut	Vein	Mgt 13	42° 11′ 48.48′' N	1° 02′ 14.21′' E
(MGT)	Vein	Mgt 14	42° 11′ 50.27′' N	1° 01′ 15.15′' E
	Strike and oblique slip faults	Mgt 15	42° 11′ 49.74′' N	1° 01′ 15.93′' E
	Strike slip fault	Mgt 20	42° 11′ 46.98′' N	1° 01′ 12.90′' E
	Normal faults	Mgt 21 – Mgt 22	42° 11′ 46.19′' N	1° 01′ 13.75′' E
	Normal fault	Mgt 23	42° 11′ 51.71′' N	1° 01′ 29.22′' E
	Strike slip and normal faults	Mgt 24 – Mgt 26	42° 11′ 51.71′' N	1° 01′ 29.22′' E
	Strike slip faults	Mgt 27 – Mgt 35	42° 10′ 50.86′' N	1° 02′ 35.83′' E

Coll de Nargó	Strike slip faults	Cn 1 – Cn 59	42° 10′ 38.81′' N	1° 18′ 13.41′' E
(CN)	Veins

**Table 2:** U-Pb ages obtained for the different fracture-filling calcite veins in the Bóixols-Sant Corneli anticline. Ages are arranged in chronological order.

Locality	Description	Sample	Age (Ma)	±2σ	MSWD	Upper intercept	Number of spots
MGT	Normal fault	Mgt21a	79.8	1.2	1.3	0.7982 ± 0.1746	20
ABC	Normal fault	Abc34	67.0	0.7	1.3	0.8227 ± 0.0041	12
SJ	Vein	Bx47a	67.1	2.2	10.6	0.8118 ± 0.0160	20
SJ	Vein	Bx47	65.4	1.3	1.3	0.8157 ± 0.0070	20
CM	Bed-parallel vein	Abc24	61.2	21.8	1.5	0.8415 ± 0.038	25
MGT	Strike-slip fault	Mgt35a	58.7	1.1	1.0	0.8213 ± 0.0157	20
ABC	Bed-perpendicular vein	Abc22	56.9	1.4	1.3	0.8261 ± 0.0239	20
MGT	Normal fault	Mgt24	48.8	8.6	0.9	0.8449 ± 0.0253	24
MGT	Strike-slip fault	Mgt15	45.5	0.8	1.4	0.8341 ± 0.0072	21
MGT	Normal fault	Mgt21b	45.3	2.5	0.4	0.8074 ± 0.0568	20
MGT	Normal fault	Mgt3	43.9	0.7	1.2	0.7629 ± 0.0598	20
OC	Vein	Bx26	43.9	1.0	1.5	0.8446 ± 0.0050	21
SJ	Strike-slip fault	Bx46	43.4	3.0	1.0	0.8232 ± 0.0064	20
MGT	Strike-slip fault	Mgt15a	42.1	2.6	1.4	0.8521 ± 0.0102	20
MGT	Strike-slip fault	Mgt20	37.8	3.5	1.2	0.8288 ± 0.0043	19
ABC	Thrust fault	Abc3	36.6	7.9	0.78	0.8055 ± 0.0145	21
ABC	Normal fault	Abc32	33.2	0.8	0.9	0.8219 ± 0.0098	20
MGT	Strike-slip fault	Mgt35b	27.6	2.3	0.5	0.8061 ± 0.0438	21
OC	Vein	Bx28	27.4	0.9	1.3	0.8389 ± 0.0074	20
MGT	Normal fault	Mgt3	20.8	1.2	1.2	0.8143 ± 0.0059	18
MGT	Normal fault	Mgt2	18.1	0.5	1.3	0.8392 ± 0.0087	9
MGT	Normal fault	Mgt1	16.8	0.2	1.6	0.8115 ± 0.0815	15
MGT	Strike-slip fault	Mgt33	9.0	4.6	0.4	0.8134 ± 0.0025	21

**Table 3:** δ^18^O, δ^13^C, ^87^Sr/^86^Sr of calcite veins and host rocks. The elemental composition, including Sr and Mn contents as well as the Y/Ho ratios, is also given (the whole elemental and REE composition is provided in the Repository). *n* corresponds to the number of analyzes. Data arranged according to the three calcite groups (geochemical trends).

			δ^18^O(‰VPDB)		δ^13^C(‰VPDB)			^87^Sr/^86^Sr			Sr (ppm)		Mn (ppm)		Y/Ho	
Locality - Description Group		n	min	max	min	max	n	min	max	n	min	max	min	max	min	max
SA - Normal fault	1	2	-5.2	-5	+2.1	+2.2	1	0.707669		2	3240	3719	175.5	178	78.5	102.7
SA - Strike slip fault	1	2	-5.1	-2.8	+2.1	+2.3	1	0.707667		1	3904		164.2		74.7	
SA - Slip surface	1	2	-4.4		+2.2		2	0.707512	0.707699	2	2070	3916	83.5	164.2	80.8	90.5
CM - Normal fault	1	5	-8.7	-5.6	+1.9	+2.1	1	0.707333		1	1213		95		81.7	
CM - Slip surface	1	4	-7.4	-6.5	+2	+2.1	1	0.707389		2	636.7	2618	79.7	84	71.5	86
CM - Vein	1	3	-9	-6.7	+2	+2.2	1	0.707285		2	2119	4689	59	72.2	71.4	90.2
ABC - Vein	1	3	-9.3	-7.9	+2.5	+2.8				2	1131.8	1381	38.1	52.5	57.5	64
CM - Vein	1	3	-10.7	-5.8	+1.4	+2.4	1	0.707355		1	2835		78.9		74.7	
CM - Vein	1	2	-9.6	-7.3	+2.2	+2.4				1	4630		82.8		74.9	
CM - Strike slip fault	1	1	-9.3		+2.2		1	0.707346		1	4746		43		84.1	
ABC - Normal fault	1	4	-9.3	-7.5	+2.3	+2.7				1	1463		56.5		75.5	
B - Normal fault	2	3	-10.3	-5.1	+2.4	+2.7	1	0.707627		3	304	708.2	89.5	150	69.2	78
MGT - Normal fault	2	7	-14.5	-9.1	+1.7	+2.5	1	0.707542		1	749		166.5		52.9	
SET - Thrust fault	2	7	-14.7	-10.4	+0.3	+1.9	2	0.707857	0.708028	2	408.7	711.5	43	82.8	52.2	60.3
CN - Vein	2	4	-13.2	-11.1	+0.4	+1.2	1	0.707468		1	794.7		319.5		53.4	
ABC - Normal fault	2	1	-7.8		-1.4					1	581.6		66.3		56.2	
OC - Vein	2	3	-13.9	-11.9	-1.8	-1	1	0.707835		1	1421		477.2		62.7	
OC - Vein	2	1	-13.1		-2.1		1	0.707894		1	1161		512		61.5	
MGT - Strike slip fault	2	1	-12.1		+1.2		1	0.707615		1	503		51.4		55.2	
FB - Vein	2	4	-13	-11.7	-3.2	-1.9	1	0.707698		3	356.2	906.7	380.5	661	44.7	51.6
FB - Thrust fault	2	6	-13.1	-11.9	-0.5	+2.4	2	0.707715	0.707771	3	626.6	670	65.2	97.3	49.9	69.6
ABC - Thrust fault	2	5	-13.8	-10.8	+1.4	+2										
OC - Vein	2	2	-12.9	-9.3	-2.8	-2.7	1	0.707920		2	530.2	805	431	467.4	54.6	60.1
MGT - Strike slip fault	2	5	-10.8	-8.8	+1.4	+2.2				1	1813.4		174.3		45	
SJ - Strike slip fault	2	6	-11.7	-8.5	-1.3	+0.9	1	0.707807		2	413	446	90.1	466.4	76.6	77.8
CN - Strike slip fault	2	15	-14.3	-9.9	-12.5	-5	2	0.707586	0.707612	2	391	519.7	119.3	186	47	52
MGT - Normal fault	2	7	-9	-8.4	+1.8	+2.6										
MGT - Strike slip fault	2	3	-13.1		+0.2											
OC - Vein	2	1	-13.8		-3.7		1	0.708018		1	707		320		56.4	
SJ - Normal fault	2	11	-13	-5.6	-2.5	+0.9	1	0.707683		2	314.6	362.5	93.2	636.2	48.2	65.2
MGT - Normal fault	2	6	-11.2	-10	+2	+2.6										
MGT - Normal fault	2	7	-13	-10.2	-4.1	-1.2	1	0.707700		2	338.7	1348	74.5	196.5	50.8	54.2
CN - Vein	3	3	-8.3	-7.8	-10.4	-8.3	2	0.707614	0.707706	2	389.4	449.8	501	1380	46.9	47.6
SJ - Vein	3	3	-8.5	-7.3	-8.2	+1.3	1	0.707762		2	164.5	441	116.5	318.4	52.3	57
CN - Vein	3	6	-9.2	-6.6	+1	+2.7	2	0.707298	0.707326	2	240.6	501.2	46.6	225.3	46.3	47.8
FB - Vein	3	5	-8.2	-6.5	-2	-1.5	1	0.707707		1	239		236.7		42.2	
FB - Vein	3	7	-8.2	-5.4	-6.3	-3.3	3	0.707695	0.707765	3	216.2	270.7	86.6	222.3	45.3	47.6
SET – Jurassic host rock		2	-8.7	-6.2	+0.7	+1.7				1	995.5		60.1		69.6	
B – Setcomelles Mb.		1	-1.6		+2.97		1	0.707530		1	722.7		104		54.4	
CM – Lluçà Fm.		1	-4.9		+2.1		1	0.707329		1	3352		80.5		60.6	
CN - Lluçà Fm.		4	-5	-3	+1.7	+2.5	1	0.707317		1	1813		76.4		70.3	
FB - Santa Fe Fm.		2	-6.1	-5.8	+2.1	+2.2	1	0.707718		1	468.9		68.8		55.7	
SJ - Congost Fm.		3	-5.5	-4.5	+1	+2.4				1	682		110		46.9	
FB - Collada Gassó Fm.		3	-7	-6.6	-0.5	+0.7	1	0.707606		1	340.8		345.6		38.8	
MGT - Sant Corneli Fm.		4	-5.6	-3.6	+2.2	+2.6										
SA & ABC - Vallcarga Fm.		2	-3.5	-2.6	+2.4	+2.8	1	0.707695		2	2643	2909	48.3	135.9	48.6	50.3
OC - Areny Group		3	-10.2	-7.8	-5	-1.9				1	1382.4		325		70.1	
CN - Garumnian		2	-7.7	-6.8	-13.1	-11				2	771.7	1362	58.5	329	50.4	54

**Table 4:** New clumped isotope results. G represents the geochemical group. n is the number of replicate measurements of the same carbonate powder. Δ_47_CDES90 are values relative to the carbon dioxide equilibrium scale (CDES) without acid fractionation correction. Paleotemperatures calculated using the composite Δ_47_-T calibration of [5] δ_18_O of water calculated using the equation of [6].

Locality	Description	G	Sample	n	δ^18^O‰ VPDB	δ^13^C‰ VPDB	Δ_47_CDES90	Δ_47_ error(‰) - SD	T (Δ_47_) °C	δ^18^O_water_‰SMOW
ABC	Bed-parallel vein	1	Abc24	3	-8.99 ± 0.07	2.25 ± 0.02	0.404	0.022	116 ± 10	6.5
SA	Normal fault	1	Bx34	2	-4.38 ± 0.00	2.14 ± 0.02	0.493	0.021	66 ± 6	5.2
SA	Bed-parallel vein	1	Bx35b	3	-4.46 ± 0.09	2.14 ± 0.02	0.475	0.024	75 ± 7	6.3
SA	Strike-slip fault	1	Bx33	3	-3.27 ± 0.09	2.23 ± 0.14	0.509	0.015	59 ± 4	5.3
CM	Normal fault	1	Bx12B	2	-6.26 ± 0.02	2.00 ± 0.04	0.487	0.016	69 ± 5	3.8
CM	Bed-parallel vein	1	Bx18	3	-6.39 ± 0.06	1.82 ± 0.03	0.502	0.017	62 ± 4	2.6
CM	Strike-slip fault	1	Bx13	3	-9.09 ± 0.05	2.02 ± 0.02	0.552	0.008	42 ± 2	-3.4
CM	Vein	1	Bx16	2	-10.31 ± 0.04	1.60 ± 0.04	0.456	0.000	85	1.7
CM	Strike-slip fault	1	Bx40	2	-9.38 ± 0.02	2.27 ± 0.00	0.567	0.028	36 ± 5	-4.7
SJ	Strike-slip fault	2	Bx19	3	-11.62 ± 0.12	0.31 ± 0,02	0.528	0.009	51 ± 2	-4.4
SJ	Normal fault	2	Bx64	1	-5.73	-2.42	0.529		50	1.5
ABC	Thrust fault	2	Abc3B	2	-14.03 ± 0.39	1.70 ± 0.06	0.475	0.022	75 ± 6	-3.3
B	Normal fault	2	Bx10	2	-7.87 ± 0.08	2.48 ± 0.06	0.522	0.022	53 ± 6	-0.1
MGT	Strike-slip fault	2	Mgt31	2	-10.59 ± 0.08	-4.96 ± 0.18	0.413	0.009	110 ± 4	4.2
MGT	Normal fault	2	Mgt4	2	-9.99 ± 0.12	2.07 ± 0.10	0.472	0.024	76 ± 7	1.0
MGT	Normal fault	2	Mgt9	2	-11.87 ± 0.05	-3.69 ± 0.08	0.470	0.013	77 ± 4	-0.8
OC	Vein	2	Bx25I	1	-12.47	-0.99	0.417		108	2.1
OC	Vein	2	Bx25II	3	-12.37 ± 0.11	-1.87 ± 0.02	0.442	0.038	92 ± 12	0.5
OC	Vein	2	Bx27	2	-9.67 ± 0.17	-2.60 ± 0.01	0.481	0.041	72 ± 9	0.7
OC	Vein	2	Bx28	3	-13.69 ± 0.19	-3.61 ± 0.04	0.477	0.023	74 ± 6	-3.1
SJ	Vein	3	Bx47	2	-7.57 ± 0.03	-3.08 ± 0.18	0.494	0.016	66 ± 5	1.9

Twenty-three new U-Pb ages were obtained by applying LA-ICPMS on 447 spot analyzes from different sets of fracture-filling calcite cements (Table 2 and Fig. 1). Obtained ages range from Late Cretaceous (79.8 ± 1.2 Ma) to late Miocene (9.0 ± 4.6 Ma). Concordia plots, which are presented in the Repository, show well-defined regression lines for most samples with mean square weighted deviation (MSWD) of < 2. An exception is sample Bx47a that has a MSWD of 10.6. As this value is higher than 2, it could indicate an open system, a mixing of ages, or an incomplete equilibration of lead isotopes [4]. The raw data of the U-Pb results are presented in the supplementary material of [1] and in the Repository.

The geochemical results, including δ^18^O, δ^13^C, ^87^Sr/^86^Sr, Δ_47_, and the elemental composition of the different fracture-filling calcite cements and host rocks are shown in Tables 3, 4 and Fig. 2. In order to summarize this robust dataset and to describe the geochemical data, the analyzed vein cements have been assembled in three calcite groups (Group 1 to Group 3) according to three observed geochemical trends:

The first geochemical trend (Group 1 calcites) has been observed in all cements from fractures and faults present in the hinge of the anticline (Cal Mestre locality) and in the base of the syn-orogenic deposits in the footwall of the Bóixols thrust sheet (Sant Antoni locality). The geochemistry of these calcites reflects the composition of their host rocks, either the Lower Cretaceous marls of the Lluçà Formation or the Upper Cretaceous marls of the Vallcarga Formation, respectively. Thus, Group 1 calcites yield minimum and maximum δ^13^C values between +1.3 and +2.4‰VPDB, respectively, and ^87^Sr/^86^Sr between 0.707285 and 0.707669, which are similar values to their adjacent host carbonates. Also, Group 1 calcites display δ^18^O values that are lower than -3 ‰VPDB and that are up to 5‰VPDB lower than those values of their adjacent rocks (Table 3). Regarding the elemental composition, these calcites have Mn contents lower than 200 ppm, Sr contents higher than 1100 ppm, and Y/Ho ratios higher than 50. Besides, nine representative samples of Group 1 cements were analyzed for clumped isotope measurements to reconstruct the composition and precipitation temperature of their parent fluids. In the hinge of the Bóixols-Sant Corneli anticline (*n* = 5), the Δ_47_ values vary from 0.456 to 0.567 ± 0.028, which indicates precipitation temperatures from 36 ± 5 to 85 °C and δ^18^O_fluid_ varying from -4.7 to +3.8‰SMOW. In the base of the syn-orogenic deposits from the footwall of the Bóixols thrust sheet (*n* = 4), the obtained Δ_47_ values, ranging from 0.404 ± 0.022 to 0.509 ± 0.015, translate into precipitation temperatures from 59 ± 4 to 116 ± 10 °C and δ^18^O_fluid_ from +5.3 to +6.5‰SMOW (Table 4).

The second geochemical trend (Group 2 calcites) has been observed in all calcite cements from large-scale faults including large thrusts, strike slip and normal faults and related fractures cutting the Bóixols-Sant Corneli anticline. These cements yield the lowest δ^18^O values, between -14 and -8‰VPDB, which are up to 10 ‰VPDB lower than those values of their host carbonates. Additionally, Group 2 calcites yield variable enrichment in δ^13^C values and ^87^Sr/^86^Sr ratios, from -12 to +2‰VPDB, and from 0.7074 to 0.7080, respectively (Table 3). Finally, comparing all calcites, Group 2 cements have intermediate Mn contents (less than 700 ppm), intermediate Sr contents (390–2000 ppm) and intermediate Y/Ho ratios (40–80). Besides, eleven representative samples of Group 2 calcites were analyzed for clumped isotope measurements to reconstruct the composition and temperature of the precipitating fluids. Obtained Δ_47_ results vary between 0.413 ± 0.009 and 0.529, which translate into δ^18^O_fluid_ from -4.4 to +4.2 ‰SMOW and temperatures from 50 to 110 ± 4 °C (Table 4).

The third geochemical trend (Group 3 calcites) has been observed in cements that precipitated in centimetric to metric-scale fractures (i.e., veins) in both limbs of the Bóixols-Sant Corneli anticline. These cements exhibit a narrow range of δ^18^O values, from -8 to -6 ‰VPDB, and tendency towards δ^13^C-depleted values, from -10 to +2‰VPDB. The ^87^Sr/^86^Sr ratios of Group 3 calcites, ranging from 0.7073 to 0.7077, are also lower than those values of their host carbonates (the Collada Gassó and the Congost Formations and the Garumnian facies) (Table 3). Finally, regarding the elemental composition, these cements have the lowest Sr contents and Y/Ho ratios, less than 500 ppm and less than 60, respectively. Besides, a representative sample of Group 3 cements was analyzed for clumped isotope measurements. The obtained Δ_47_ values, which are 0.494 ± 0.016, translate into precipitation temperatures of 66 ± 5 °C and δ^18^O_fluid_ of +1.9‰SMOW (Table 4).

## 2 Experimental Design, Materials and Methods

Petrographic analysis of around 135 polished thin sections from host rocks and vein cements was made using a Zeiss Axiophot microscope and a cold cathodoluminescence (CL) microscope operating at 15–18 kV and 350 µA current.

U-Pb ages were obtained with a laser ablation-inductively coupled plasma mass spectrometer (LA-ICPMS) at FIERCE (Frankfurt Isotope and Element Research Center, Goethe University), following the modified method of [7]. A Thermo Scientific Element XR sector field ICPMS was coupled to a RESOlution 193 nm ArF excimer laser (COMpexPro 102) equipped with a two-volume ablation cell (Laurin Technic S155). Samples were firstly ablated in a helium atmosphere (300 mL/min) and then mixed in the ablation funnel with 1100 mL/min argon and 5 mL/min nitrogen. Signal strength at the ICP-MS was tuned for maximum sensitivity but keeping the oxide formation (monitored as ^248^ThO/^232^Th) below 0.2% and low fractionation of the Th/U ratio. Static ablation used a spot size of 193 μm and a fluency of about 2 J/cm^2^ at 12 Hz.

Data were obtained in fully automated mode overnight in two sequences of 598 analyzes each one. Each analysis comprised 18 s of background acquisition, 18 s of sample ablation, and 25 s of washout. During 36 s of data acquisition, the signal of ^206^Pb, ^207^Pb, ^208^Pb, ^232^Th, and ^238^U was detected by peak jumping in pulse-counting and analogue mode with a total integration time of _∼_0.1 s, resulting in 360 mass scans. Each spot was pre-ablated with 8 laser pulses to remove surface contamination before analysis. Soda-lime glass NIST SRM-612 was used as primary reference material (spot size of 50 μm, 8 Hz) together with four carbonate reference materials, which were bracketed in between the analysis of samples.

Raw data were corrected offline with an in-house VBA spreadsheet program [7]. Following background correction, outliers (±2σ) were rejected based on the time-resolved ^207^Pb/^206^Pb, ^208^Pb/^206^Pb, ^206^Pb/^238^U, and ^232^Th/^238^U ratios. Such ratios were corrected for mass biases and drift over time, using NIST SRM-612. An additional matrix related offset was applied on the ^206^Pb/^238^U ratios (sequence 1: 21.5%, sequence 2: 19.6%) that was determined using WC-1 carbonate reference material [8]. The ^206^Pb/^238^U downhole-fractionation was estimated to be 3%, based on the common Pb corrected WC-1 analyzes, and was applied to all carbonate analyzes. Uncertainties for each isotopic ratio are the quadratic addition of the within run precision, counting statistic uncertainties, excess of scatter (calculated from NIST SRM-612) and the excess of variance (calculated from WC-1) after drift correction [9]. The systematic uncertainties considered are the decay constants uncertainties and the long-term reproducibility of the method (1.5%, 2σ, calculated from repeated measurements (*n* = 7) of ASH-15D between 2017 and 2019).

Carbonate reference materials were measured for quality control. Reference material B6 (41.86 ± 0.53 Ma and 42.12 ± 0.88 Ma) [10] was measured in sequences 1 and 2, whereas reference material ASH-15D (2.907 ± 0.210 Ma) [11] was measured in sequence 1. Results on the secondary reference materials indicate an accuracy and repeatability of the method of about 1.5–2%. Data were displayed in Tera-Wasserburg plots, and ages were calculated as lower concordia-curve intercepts using the same algorithms as Isoplot 4.14 [12]. All uncertainties are reported at the 2σ level. Analytical results, Concordia graphs and a summary of the U-Pb results are reported in [1].

For carbon and oxygen isotopes of vein cements and carbonate host rocks, 50–100 µm of samples were extracted with a microdrill. Each powered sample was reacted during four minutes with 100% phosphoric acid at 70 °C. The resultant CO_2_ was analyzed following the method of [13] and using an automated Kiel Carbonate Device attached to a Thermal Ionization Mass Spectrometer Thermo Electron MAT-252 (Thermo Fisher Scientific). For calibration, the internal standard RC-1 (δ^13^C_VPDB_ = +2.83‰, δ^18^O_VPDB_ = -2.08‰) and CECC (δ^13^C_VPDB_ = -20.77‰, δ^18^O_VPDB_ = -17.56‰), traceable to the International Standard NBS-19 (δ^13^C_VPDB_ = +1.95‰, δ^18^O_VPDB_ = -2.20‰), and the International Standard NBS-18 (δ^13^C_VPDB_ = -5.1‰, δ^18^O_VPDB_ = -23.2‰) have been employed. Results are expressed in ‰ with respect to the Vienna Pee Dee Belemnite (VPDB). Standard deviation is ±0.04 ‰ for δ^18^O and ±0.02 ‰ for δ^13^C.

Δ_47_ measurements were performed at the California Institute of Technology (USA) in three different analytical sessions (May to July 2019) with an automated acid digestion and gas purification device coupled to a dual inlet Thermo MAT253 [14]. Samples were weighed into silver capsules (∼ 8 mg) and reacted in a common phosphoric acid bath (∼103%) for 20 min at 90 °C under static vacuum. The evolved CO_2_ was passed through an ethanol/dry ice U-trap (∼ -80 °C) before being collected on a liquid nitrogen temperature (-196 °C) U-trap. Following the 20 min reaction period, the collected CO_2_ was thawed, entrained in helium, and carried through a Porapak Q 120/80 mesh gas column held at -20 °C using He as the carrier gas. The purified CO_2_ was analyzed using a Thermo Scientific MAT 253 Mass Spectrometer set to collect masses 44–49. Mass 48 was only monitored to detect any hydrocarbon contaminant. δ^18^O and δ^13^C data were also acquired as part of each Δ_47_ analysis and calculated using the parameters reported relative to the PDB reference frame based on the calibrated composition of the laboratory working gas and the correction scheme and constants from [15]. To account for the temperature dependence of oxygen isotope fractionation between CO_2_ gas and carbonate resulting from the reaction with phosphoric acid at 90 °C, fractionation factors of 1.00811 were used for calcite [16]. The raw Δ_47_ data was corrected for instrument non-linearity and scale compression [17] using several heated (at 1000°) and equilibrated gases (at 25 °C) of various bulk isotopic compositions that were run during each session. These gases were used to convert measurements into the interlaboratory absolute reference frame [17]. To guarantee accuracy of the Δ_47_ data, we routinely analyzed two carbonate reference materials (Carrara marble and TV04). One of these two carbonate standards was analyzed typically once for every five analyzes of the unknown samples to check for procedural analytical stability and accuracy, and to determine the long-term external reproducibility of our measurements. The Δ_47_ values obtained for these carbonates over the course of this study are: Δ_47-CDES25_ = 0.409 ± 0.016‰ (1SD, *n* = 10) for Carrara; Δ_47-CDES25_ = 0.666 ± 0.011‰ (1SD, *n* = 8) for TV04, i.e., within accepted Δ_47_ values for TV04 (Δ_47-CDES25_ = 0.655‰) and Carrara (Δ_47-CDES25_ = 0.405‰). Finally, the corrected Δ_47_ values were converted into temperatures using the composite Δ_47_-T calibration of [5], which has been shown to be appropriate for calcite and dolomite between 0 and 300 °C. The oxygen isotopic compositions of the water (δ^18^O_water_) from which the carbonates precipitated were calculated for each estimated TΔ_47_ using the bulk δ^18^O_carb_ values and the calcite-water fractionation equation from [6].

For ^87^Sr/^86^Sr ratios, powdered samples of calcite cements and host rock have been dissolved in 5 mL of 10% acetic acid and then centrifuged. The supernatant was dried and dissolved in 1 mL of 1M HNO_3_. The solid residue, resulted after evaporation, was diluted in 3 mL of 3M HNO_3_ and then loaded into chromatographic columns to separate the Rb-free Sr fraction, using SrResin^TM^ (crown-ether (4,4’(5’)-di-t-butylcyclohexano-18-crown-6)) and 0.05M HNO_3_ as eluent. After evaporation, samples were loaded onto a Re filament with 2 µL of Ta_2_O_5_ and 1 µL of 1 M phosphoric acid. Analyzes of isotopic ratios have been performed in a TIMS-Phoenix mass spectrometer (Isotopx) according to a dynamic multicollection method, during 10 blocks of 16 cycles each one, maintaining a ^88^Sr beam intensity of 3-V. Obtained ratios have been corrected for ^87^Rb interferences and normalized with a ^88^Sr/^86^Sr = 0.1194 reference value, aiming at correcting possible mass fractionation during sample loading and analysis. The isotopic standard NBS-987 has been analyzed 6 times, yielding an average value of 0.710243 ± 0.000009 (standard deviation, 2σ). NBS 987 data have been used to correct the sample ratios for standard drift from the certified value. The analytical error in the ^87^Sr/^86^Sr ratio was 0.01% (referred to two standard deviations). The internal precision is 0.000003. Sr procedural blanks were below 0.5 ng.

For the elemental composition, powdered samples of vein cements and host rocks were analyzed employing a magnetic sector field Element XR (HR-ICP-MS, high resolution inductively coupled plasma-mass spectrometer, Thermo Fisher Scientific). In this case, the LR (low resolution) and the MR (medium resolution) have only been used. 100 mg of each powdered sample was firstly dried at 40 °C during 24 h and then acid digested in closed polytetrafluoroethylene (PTFE) vessels with a combination of HNO_3_+HF+HClO_4_ (2.5 mL: 5 mL: 2.5 mL v/v). Samples have been evaporated and, to make a double evaporation, 1 mL of HNO_3_ was added. Then, samples have been re-dissolved and diluted with MilliQ water (18.2 MΩ cm-^1^) and 1 mL of HNO_3_ in a 100 mL volume flask. A tuning solution of 1 µg L^−1^ Li, B, Na, K, Sc, Fe, Co, Cu, Ga, Y, Rh, In, Ba, Tl, U was employed to improve the sensitivity of the ICP-MS and 20 mg L^−1^ of a monoelemental solution of ^115^In were used as internal standard. Reference materials are the BCS-CRM n^o^ 393 (ECRM 752-1) limestone, JA-2 andesite and JB-3 basalt. Precision of results is expressed in terms of two standard deviations of a set of eight reference materials measurements (reference material JA-2). Accuracy (%) has been calculated employing the absolute value of the difference between the measured values obtained during the analysis and the certified values of a set of eight reference material analysis (reference material BCS-CRM n^o^ 393 for major oxides and JA-2 for trace elements). The DL (detection limit) has been calculated as three times the standard deviation of the average of ten blanks.

## Ethics Statement

Nothing to declare.

## CRediT authorship contribution statement

**Daniel Muñoz-López:** Conceptualization, Data curation, Formal analysis, Investigation, Writing – original draft, Writing – review & editing. **David Cruset:** Data curation, Formal analysis, Investigation. **Jaume Vergés:** Formal analysis, Investigation, Funding acquisition. **Irene Cantarero:** Data curation, Formal analysis, Investigation. **Antonio Benedicto:** Data curation, Formal analysis, Investigation. **Vinyet Baqués:** Formal analysis, Investigation. **Xavier Mangenot:** Methodology. **Richard Albert:** Methodology. **Axel Gerdes:** Methodology. **Aratz Beranoaguirre:** Methodology. **Anna Travé:** Data curation, Formal analysis, Investigation, Funding acquisition, Project administration.

## Declaration of Competing Interest

The authors declare that they have no known competing financial interests or personal relationships that could have appeared to influence the work reported in this paper.

## Data Availability

Geochronological and geochemical data of calcite veins in the Bóixols-Sant Corneli anticline (Southern Pyrenees) (Original data) (Mendeley data).
